# The impact of social isolation on physical and mental health among empty-nest older adults: an integrative review

**DOI:** 10.3389/fpubh.2026.1789519

**Published:** 2026-04-08

**Authors:** Yi Zhang, Xinyan Zhou, Yufan Wang, Min Liu, Peng Gao, Mei Ju

**Affiliations:** 1 School of Nursing, Southwest Medical University, Luzhou, Sichuan, China; 2 Department of Nursing, Affiliated Hospital of Southwest Medical University, Luzhou, Sichuan, China

**Keywords:** empty-nest older adults, integrative review, mental health, physical health, social isolation

## Abstract

**Introduction:**

To our knowledge, previously there have been no reviews about the impact of social isolation on physical and mental health among people aged over 60, who are empty-nest older adults.

**Objective:**

To conduct an integrative review of empirical studies to provide a comprehensive understanding of social isolation and its influence among empty-nest older adults' health.

**Design:**

An integrative literature review.

**Data sources:**

PubMed, Web of Science, Scopus, Embase, EBSCOhost, CNKI, Wanfang data, VIP were searched for studies from the database inception to October 2025. Of 2,833 scrutinized articles, 14 were eligible for inclusion and subjected to independent quality appraisal. One mixed-method study, two qualitative research studies, and 11 quantitative research studies were selected.

**Results:**

Social isolation among empty-nest older adults is linked to elevated physical health risks, including higher mortality, as well as mental health issues such as depression, self-neglect, loneliness, reduced life satisfaction, and cognitive decline. It also impacts comprehensive health indicators like intrinsic capacity and quality of life. Mediating factors, including aging attitudes, loneliness, perceived stress, and health-promoting behaviors, exacerbate these effects.

**Conclusion:**

Social isolation adversely affects both physical and mental health in empty-nest older adults, underscoring the need for developing interventions to promote social support networks and expand social connections.

## 1 Introduction

The global aging population has been rapidly increasing in recent years, and the United Nations (UN) has reported that the number of older persons aged 65 years or older is predicted to grow from approximately 830 million in 2024 to over 2.2 billion in the late 2070s (1). However, because of declining fertility rates, and evolving patterns in marriage and cohabitation, demographic shifts are driving the reshaping of living arrangements for the older adults, including changes in household size and composition. In 2020, a United Nations report indicates that the majority of older adults aged 65 and older live alone or with their spouse in developed countries, while in developing countries, most of them typically live with their children or kin such as grandchildren, nieces, nephews and in-laws (2). But there is some difference between genders, 23% of older women prefer to live alone, which is twice as likely as that of the men (11%). Conversely, 36.7% of older men are more likely to cohabit with a partner, which is 1.7 times as likely as that of women (22.2%) (2). Thus, these reasons lead to more older adults becoming empty-nest older adults. The definition of empty-nest older adults is not entirely clear, in this review, the empty nesters are defined as those older adults over 60 years old, who are childless or whose children are absent for a long time, living alone or with a spouse (3). Some empirical studies have demonstrated that empty-nest older adults are more vulnerable than non-empty-nest older adults in terms of physical and mental health (4, 5). Meanwhile, owing to inherent characteristics such as living arrangements and social networks, empty-nest older adults individuals may be more susceptible to social isolation.

Previous studies have shown that living arrangements were related to social isolation (6–9). Social isolation refers to an objective state characterized by a lack of social contact and interactions with friends, family members, or the community (10). It is conceptually and operationally distinct from subjective loneliness (perceived lack of companionship) (11, 12) and structural living arrangements (living alone or empty-nest status) (12), although the latter may increase exposure to SI. And it has emerged as a significant public concern for older adults in contemporary society (13, 14). A meta-analysis showed that the global prevalence of social isolation among older people is up to 35% (95% CI: 0.28–0.38), and those who were living alone and lacked higher education experienced higher risk of social isolation (15). Furthermore, additional risk factors, particularly among empty-nest older adults, include female gender (16), low socioeconomic status, poor health conditions (17), rural-urban disparities, and negative attitudes toward aging (18). For example, in China, a study indicated that the prevalence of social isolation among empty-nest older adults is significantly higher than that among non-empty-nest older adults (34.0% vs. 29.2%, *p* < 0.01) (19). Within the empty-nest older adult population, rural areas are particularly affected, with the prevalence of social isolation reaching as high as 57.3% due to the migration of adult children (17). A cohort study in Japan revealed that the prevalence of social isolation among individuals aged 65 and older is 22.0%, including 19.2% among those living alone (20). A survey in Korea targeting female older adults aged 65 and older who live alone found that the prevalence of social isolation is as high as 36.6% (21). These high prevalence rates of social isolation underscore its potential to profoundly impact the physical health and associated consequences.

Numerous studies have shown that social isolation has adverse physical and mental health impacts on older adults (22, 23). For example, social isolation is highly linked with cardiovascular events such as hypertension, stroke, and coronary heart disease (24–26). Social isolation also raises the risk of depression by 77% and anxiety by 66% [adjusted ORs: 1.77 (95% CI: 1.25–2.51) and 1.66 (95% CI: 1.05–2.63), respectively] (27). These ill effects above of social isolation further result in decline in cognitive functioning (28), which leads to poor quality of life (29) and may result in early mortality (30).

Although some studies have explored the impact of social isolation on the physical and mental health of older adults, research specifically targeting the empty-nest older adults as a distinct subgroup remains scarce. To our knowledge, no systematic review has been conducted on the impacts of social isolation on the physical and mental health among empty-nest older adults individuals. Therefore, this study employs an integrative review approach to examine prior research, aiming to explore the evidence regarding the influence of social isolation on the physical and mental health of the empty-nest older adults.

## 2 Method

The integrative review was conducted using the framework proposed by Whittemore and Knafl (31), which is particularly suitable for synthesizing evidence derived from diverse methodologies and data sources. Applying this framework to the topic of social isolation and its impact on health among empty-nest older adults, the review proceeded through five stages: (a) problem identification, (b) literature search, (c) data evaluation, (d) data analysis, and (e) presentation of results.

### 2.1 Problem identification

The aim of this integrative review was to examine the available evidence on the impact of social isolation on the health of empty-nest older adults. The research questions of this review were as follows:

1. How does social isolation affect the physical health and/or mental health of empty-nest older adults?
2. Which factors mediate or moderate the impact of social isolation on the health of empty-nest older adults?

### 2.2 Literature search

#### 2.2.1 Search strategy and eligibility criteria

Literature searches were performed in three Chinese electronic databases (CNKI, Wanfang Data, VIP) and five English electronic databases (PubMed, Web of Science Core Collection, Scopus, Embase, EBSCOhost) from inception to October 2025. In the preliminary literature search, we employed keywords such as “empty-nest”, “older adults”, “social isolation”, and “health” to conduct database retrievals. However, upon identifying a limited number of studies in the domain of social isolation among empty-nest older adults, we refrained from imposing strict restrictions on the “health” keyword in subsequent searches to ensure the completeness and comprehensiveness of the literature. Thus, search terms included but were not limited to the following keywords/MeSH terms: empty nest, older adults, social isolation, social exclusion. Terms were used alone and combined using Boolean Operators (and/or). As an example, the search string used in PubMed was: [(“empty nest” OR “empty-nest” OR “left-behind” OR “living alone” OR “solitary” OR “childless”) AND (“aged” OR “older adults” OR “geriatric” OR “older adults” OR “seniors”)]. Table 1 shows the eligibility criteria for the selected papers.

**Table 1:** SPIDER inclusion and exclusion criteria.

Domain	Inclusion criteria	Exclusion criteria
Sample	Participants were **empty-nest older adults** aged ≥60 years, defined as living alone or living only with a spouse, with no children co-residing (but including **left-behind older adults** whose adult children have migrated for work); No restrictions of empty nesters' physical, psychological or medical condition.	Participants' age are not all ≥60 years; Participants were <60 years old or co-resided with children or other relatives.
Phenomenon of interest	Studies explicitly examined the association or impact of SI (objective lack of social contacts) on any health outcome.	Studies that only focus on SI or health (physical or mental health); No information about the relation between SI and health; Studies that assessed only loneliness (subjective feeling) without measuring SI; Studies that mentioned SI only in the introduction/discussion without presenting primary data on its relationship with health.
Design	Primary research	Secondary research
Evaluation	SI using validated instruments (e.g., LSNS-6, LSNS, Lubben social network scale, Duke social support index, social network index) or researcher-defined criteria based on frequency/size of social contacts; For qualitative studies: a clear description of SI as a theme or concept; Any physical, mental, or social health-related outcome (e.g., mortality, depression, anxiety, cognitive function, quality of life, self-rated health, chronic diseases, etc.).	Loneliness scales only defined as tool to assess loneliness not SI (e.g., UCLA 3-item or 20-item).
Research type	Primary research: qualitative, quantitative or mixed-method studies; Full-text articles published in Chinese or English.	Reviews, editorials, conference abstracts, protocols, or studies without primary data.

#### 2.2.2 Selection processes

**Figure 1:**
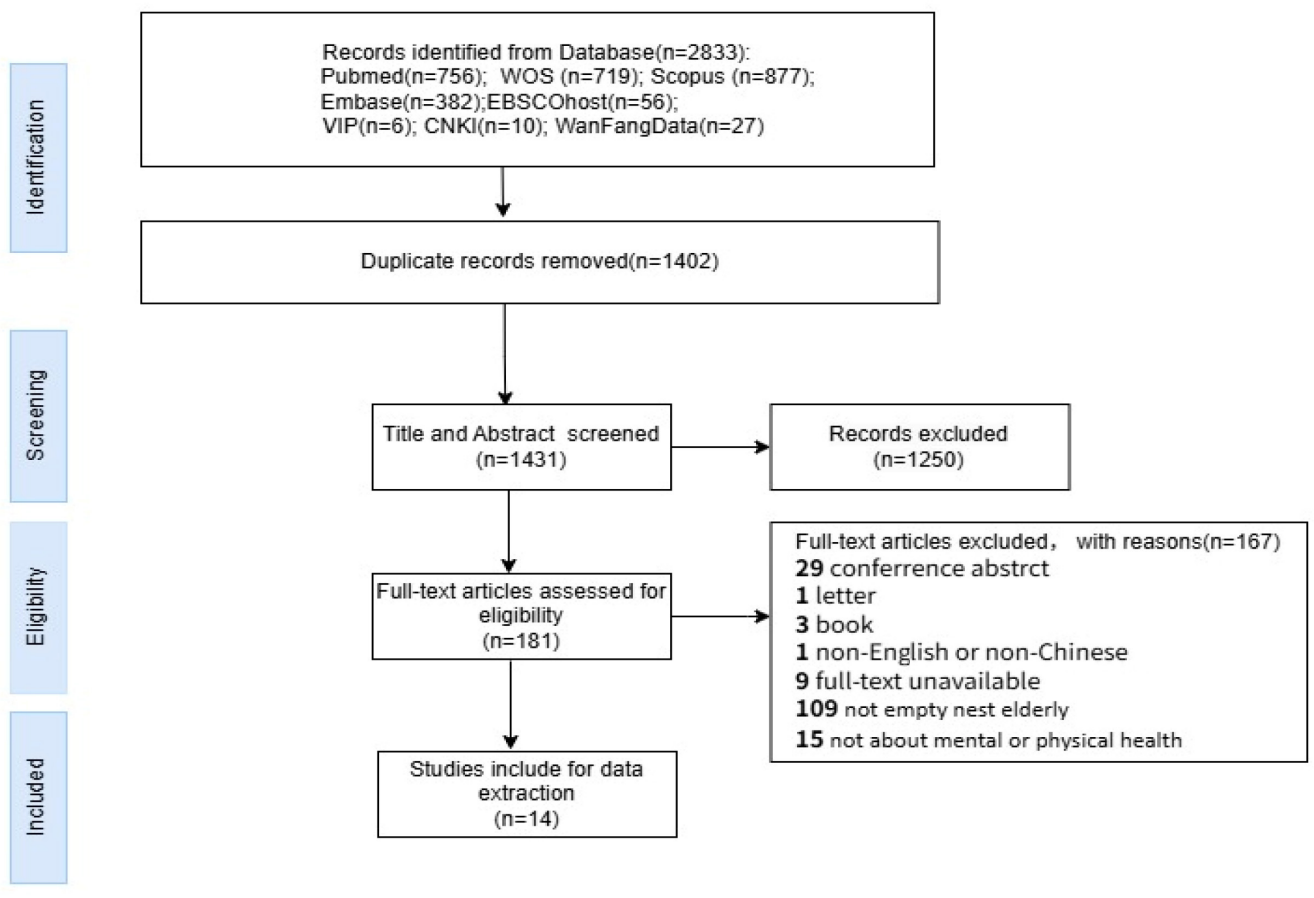
Preferred reporting items for systematic reviews and meta-analyses flow diagram of study selection.

The data search was reported in the Preferred Reporting Items for Systematic Reviews and Meta-analysis-2020 (PRISMA-2020) guidelines (32) for transparency of the search process. This hybrid approach—adopting the Whittemore and Knafl integrative review framework while following PRISMA-2020—allows synthesis of diverse methodologies and evidence while maintaining systematic and reproducible search procedures. The PRISMA flow diagram is shown in Figure 1. First, retrieved sources were imported into EndNote 21 reference management software, then removed duplicates and transformed into an Excel sheet for data screening. One reviewer (ZY) checked the Excel sheet manually to identify duplicates again. Second, two reviewers (ZY and ZXY) independently screened the title and abstracts of citations against eligibility criteria. Finally, full texts were independently screened by the two reviewers (ZY and WYF). Any discrepancies were discussed and resolved by the team. Meanwhile, the reasons for the excluded papers were recorded in Figure 1.

The results of eight database searching were 2,833 records. After removing 1,402 duplicate records, there were 1,431 records left for the review of titles and abstracts. Based on exclusion criteria, 1,250 records were further removed. As a result, 181 records left that were eligible for full-text screening. However, 167 of them were removed as they were either conference abstract, letter, book, non-English or non-Chinese, unavailable in full-text, not empty nest older adults related or not about mental or physical health. Finally, only 14 articles were included in the integrative review.

### 2.3 Data evaluation

The Mixed Methods Appraisal Tool (MMAT) (33), which is specifically designed to evaluate quantitative studies, qualitative studies and mixed-methods research, was used to assess the methodological quality of included studies. Data were evaluated for methodological rigor and data relevancy by two reviewers (ZY and ZXY) independently, and consensus was reached following discussion.

### 2.4 Data analysis

Given the methodological heterogeneity of the included studies (quantitative, qualitative, and mixed-methods designs), a narrative synthesis was undertaken for the included papers. The first researcher (ZY) performed the analysis, and the other two researchers (ZZY and GP) checked and verified the results for accuracy. Discrepancies were discussed to reach a consensus.

In the current study, data reduction involved extracting and categorizing key details, such as author, year, country, sample, study design, measurement tools, and main results regarding health. Then, the data display was organized into themes and displayed in a table. What's more, quantitative results were descriptively compared, while qualitative findings underwent thematic synthesis via constant comparison. Finally, the findings in the current review were compared with the primary sources to maintain accuracy and conformability.

### 2.5 Presentation

Text and tables were used to present the data narratively.

## 3 Results

Considerable heterogeneity existed in the operationalization and measurement of social isolation. Seven studies employed validated social network scales (primarily the LSNS-6), two used frequency-of-contact questions, one adopted an adapted Berkman Social Network Index, one relied on broader social exclusion indicators, one mixed-methods study assessed social isolation via semi-structured interviews inquiring about contact quantity and frequency (face-to-face or telephone) as well as confidants for intimate concerns, and two qualitative studies treated social isolation as an emergent theme (see Table 2 for details). Despite these variations in measurement approaches, the direction and statistical significance of the associations with physical and mental health outcomes remained largely consistent across different measurement methods, thereby further strengthening the robustness of the synthesized findings.

**Table 2:** Summary of social isolation measurement tools in included studies.

Measurement type	Author, reference	Specific Tool/Operationalization	Cut-off	Explicit distinction from living alone/loneliness
Standardized Validated Network Scales	Jia et al. (35)	Lubben Social Network Scale-6 (LSNS-6)	Total score <12	Yes (living alone is sampling background; loneliness treated as separate construct)
	Jia et al. (8)			Yes
	Jia et al. (18)			Yes
	Gu et al. (39)			Yes
	Guo et al. (40)			Yes
	Su et al. (41)			Yes
	Xie et al. (42)			Yes
Objective Contact Frequency Criteria	Imamura et al. (20)	Frequency of face-to-face and non-face-to-face contact (telephone, mail, etc.) with non-cohabiting family/relatives/friends/neighbors	<1 time per week for both types of contact	Yes (explicitly independent of living alone; analyzed separately and jointly)
	Imamura et al. (43)			Yes (explicitly distinguished from living alone)
Custom/Adapted Measures	Tong et al. (34)	Five items within the “social relations” dimension of a social-exclusion framework (telephone contact, in-person meeting, someone to trust, help when unable to walk, loneliness reverse-scored)	No single cut-off (continuous scoring)	Yes (the loneliness (1 item) and the relative social isolation (4 items) are calculated separately; living alone is sampling criterion)
	Hwang and Son Hong (44)	Adapted Berkman Social Network Index (SNI; 3 items: religious activity frequency, group/club participation, number of close contacts)	SNI score 0–1 = social isolation	Yes (marital status excluded; independent of living alone)
Qualitative Narrative	Melchiorre et al. (36)	Semi-structured interview exploring network size, contact frequency/mode (face-to-face/telephone), and confidants for intimate concerns or support	No quantitative cut-off	Yes (explicitly distinguished from loneliness and functional social support)
	Kwan and Tam (37)	Qualitative thematic analysis of lived experiences	None	Partial (focuses on living-alone experiences; isolation implied)
	Soulières and Charpentier (38)	Qualitative interviews with conceptual definition (limited interpersonal relationships, social roles, and community participation)	No quantitative tool	Yes (emphasizes objective measurable situation, distinct from subjective experience)

### 3.1 Characteristics of studies

The search strategy identified a total of 2,833 articles. Among them, 14 studies were included in the review, including 11 descriptive quantitative studies (10 cross-section studies and 1 longitudinal study), 2 qualitative studies, and 1 mixed-method research. A total of 9,175 participants aged over 60 years were included, with 9,085 participants involved in 11 quantitative studies, 90 in two qualitative studies and 120 in one mixed study. Among them about 5,217 participants could be categorized as empty-nest older adults (Figure 1). Table 3 shows the main characteristics of the included studies. According to the analysis, 7.1% (1/14) of the studies were published in 2011, 7.1% (1/14) in 2020, 21.4% (3/14) in 2022, 7.1% (1/14) in 2023, 35.7% (5/14) in 2024, and 21.4% (3/14) in 2025. Regarding the origin, 64.3% (9/14) of studies on this topic in China were conducted, 14.3% (2/14) in Japan, and a lower rate of 7.1% (1/14) studies was found in following countries: Canada, Italy, Korea.

**Table 3:** Summary of included articles (*n* = 14), considering year and country of the study, aims, design, sample, outcome measures, main results.

Author (references) Country	Aims	Design	Sample	Outcome measures	Main results
Jia et al. (35) China	To examine self-neglect profiles, characteristics, and associated factors among rural older adults living alone.	**Cross-sectional study** Convenience sampling method From December 2022 and February 2023.	*N* = 499 *M* = 199 (39.9%) *F* = 300 (60.1%) Aged ≥ 60 years Living alone ≥3 months.	**LSNS-6;** SESN; Family APGAR	Three distinct self-neglect categories were identified: “low self-neglect-high medical healthcare self-neglect” (59.32%), “high overall self-neglect” (18.64%), and “high emotional self-neglect” (22.04%). Gender, ethnicity, family functioning, and SI were associated with self-neglect profiles. SI (no as reference) was significant in the high overall self-neglect group (OR = 3.596, 95% CI: 1.979–6.532) and the high emotional self-neglect group (OR = 2.268, 95% CI: 1.27–4.051)
Jia et al. (8) China	To investigate the relationships among aging attitudes, SI, and self-neglect, and how aging attitudes mediate between SI and self-neglect among rural older adults living alone in China.	**Cross-sectional study** Convenience sampling method From December 2022 and February 2023.	*N* = 499 *M* = 199 (39.9%) *F* = 300 (60.1%) Aged ≥ 60 years Living alone ≥3 months.	**LSNS-6;** SESN; AAQ	SI was positively associated with aging attitude (*r* = 0.353, *P* < 0.05) and negatively correlated with self-neglect (*r* = −0.371, *P* < 0.05). Self-neglect was also negatively correlated with aging attitude (*r* = −0.367, *P* < 0.05). Aging attitude partially mediated the relationship between SI and self-neglect among older adults living alone in rural areas, with a mediation effect of −0.077, accounting for **28.20%** of the total effect.
Jia et al. (18) China	To examine the interrelationships between social networks, loneliness, and self-neglect, focusing on the mediating role of loneliness.	**Cross-sectional study** Convenience sampling method From December 2023 and February 2024.	*N* = 582 *M* = 202 (34.7%) *F* = 380 (65.3%) Aged ≥ 60 years Living alone ≥6 months.	**LSNS-6** SESN; ULS-6	SI prevalence reached 38.5%, with 90% reporting loneliness. Correlation analysis showed that social network was negatively correlated with loneliness (*r* = −0.476, *p* < 0.05) and self-neglect (*r* = −0.421, *p* < 0.05), while loneliness was positively correlated with self-neglect (*r* = 0.455, *p* < 0.05). Loneliness partially mediated the relationship between social network and self-neglect, accounting for **37.22%** of the total effect.
Gu et al. (39) China	To investigate the prevalence of depression and identify its associated factors among community-dwelling older adults living alone in China.	**Cross-sectional study** Convenience sampling method From September 2017 to April 2018	*N* = 172 *M* = 62 (36.0%) *F* = 110 (64.0%) Aged ≥ 60 years Living alone for ≥12 months.	**LSNS-6** GDS-15; SPMSQ; Lawton and Brody ADL	The prevalence of depression among this group of Chinese older adults was 18.6%. The possibility of depression among older adults who had SI risk was 2.59 times higher than those who had no such risk.
Guo et al. (40) China	To examine intrinsic capacity (IC) subgroups and the association of IC subgroups with IC predictors in Chinese urban empty nesters.	**Cross-sectional study** Convenience sampling method From June to December 2023.	*N* = 385 Age ≥ 60 years *F* = 166 (43.1%) *M* = 219 (56.9%) Community empty-nest older adults (living in the community ≥6 months, and not living with their children).	**LSNS-6** ICOPE; SESN; ULS-6	There are three IC subgroups: “Low IC level Low locomotion domain”(33.5%o), “Medium IC level-Low sensory domain” (16.9%o) and “High IC level” (49.6%o). Age, marital status, receiving visits from children, multimorbidity, **social network**, loneliness, and self-neglect are factors correlated with IC. Community empty-nest older adults in the “Low IC Level—Low locomotion” class have the lowest overall social network score, followed by the “Medium IC level—Low sensory domain” class, and the “High IC Level” class has the highest social network score.
Su et al. (41) China	To explore the relationship between SI, perceived stress, health promotion behavior, and IC of the left behind older adults in rural areas and analyzes the chain mediating effect of health promotion perceived stress and behavior among SI and IC.	**Cross-sectional study**, multi-stage sampling method From March 2021 to May 2022.	*N* = 366 *M* =166 (45.4%) *F* =200 (54.6%) Aged ≥ 60 years Rural **left-behind** older adults **recruited from the wards**, whose offspring had been working outside for >6 months.	**LSNS-6;** CPSS; HPLP-C; ICOPE	SI was positively correlated with health-promoting behavior (r = 0.78, p < 0.01) and IC (r = 0.67, p < 0.01), whereas SI was negatively correlated with perceived stress (r = −0.63, p < 0.01). Perceived stress was negatively correlated with health-promoting behavior (r = −0.62, p < 0.01) and IC (r = −0.43, p < 0.01). Finally, there was a correlation between health promoting behavior and IC (r = 0.56, p < 0.01). Bootstrapping values indicated that the chain-mediating effect of perceived stress and health-promoting behavior was statistically significant.
Xie et al. (42) China	To investigate quality of life and its associated factors among community-dwelling oldest old persons who live alone.	**Cross-sectional study** Convenience sampling method From May to July 2021.	*N* = 218 *M* = 38 (17.43%) *F* = 180 (82.57%) Aged ≥ 75 years Living alone ≥6 months.	**LSNS-6** WHOQOL-OLD; FRAIL scale; GDS-5; ULS-6;	The mean overall WHOQOl-OLD score for the participants was (60.21+13.68), domains of social participation (60.21+13.68), sensory abilities (55.44+21.73), and intimacy (53.93+22.20) had the lowest mean scores. Results of linear regression model showed that age, polypharmacy, physical frailty, depression, loneliness, and SI were independently associated with overall WHOQOL-OLD score, accounting for 52% of the variance. The proportion of variance explained by loneness, **SI**, depression, physical frailty, age and polypharmacy on the overall WHOQOL-OLD scores was 13.06%, **12.38%**, 9.74%, 6.67%, 3.59% and 3.27% respectively.
Imamura et al. (20) Japan	To investigated the combined associations of SI and living alone with mortality among community-dwelling older adults.	**Longitudinal study** Census sampling method September 25–October 5 2012 or October 7–18 2013; follow-up: November 1 2020.	*N* = 1106 *M* = 459 (41.5%) *F* = 647 (58.5%) Aged ≥ 65 years No SI nor living alone = 653; No SI but living alone = 210; SI but not living alone = 193; SI and living alone = 50.	**SI:** asking **how often** they interacted with **family**, **relatives, friends, and neighbors** SRH; IADL; ZSDS; MMSE; number of comorbidities; usual gait speed; all-cause mortality.	There are 50 participants (4.5%) experienced both SI and living alone. This combination was associated with a worse prognosis regarding all-cause mortality (HR = 2.08, 95% CI:1.08-4. 00), while the participants of SI but not living alone also showed a trend toward higher mortality risk (HR = 1.41, 95% CI: 0.90-2.20). Contrastingly, those who were not socially isolated and lived alone did not show an increased mortality risk (HR = 0.81, 95% CI: 0.44-1.49).
Imamura et al. (43) Japan	To examine the association of SI and living alone with cognitive impairment in community-dwelling older adults.	**Cross-sectional study** Data from the integrated research initiative for living well with Dementia Cohort Study (2010–2018).	*N* = 4,362 *M* = 1,932 (44.3%) *F* = 2,430 (55.7%) Aged ≥ 65 years No SI nor living alone = 2,139; no SI but living alone = 844; SI but not living alone = 973; SI and living alone = 406.	**SI**: asking the frequency of interactions with **family members, relatives, friends, or neighbors**. MMSE.	Of the 4,362 participants included in the analysis (mean age 75.6 years, 44.3 % male), 11 % had cognitive impairment. Regardless of living alone, SI was associated with cognitive impairment (no SI x not living alone: reference, SI x not living alone; OR: 1.74, 95 % CI: 1.29-2.33, **SI x living alone; OR: 2.10, 95 % CI: 1.46-3.01**).
Tong et al. (34) China	To examined the effects of social exclusion on depressive symptoms in older Chinese who are living alone in China, based on the data from one Shanghai neighborhood.	**Cross-sectional study** Simple random sampling method from August to October 2008.	*N* = 228 *M* = 63 (27.6%) *F* = 165 (72.4%) Aged ≥ 60 years Living alone rural: 34.7% urban: 65.3%	**Social exclusion**: includes adequacy of income, housing conditions, **social relations (SI and Loneliness)**, civic participation, and use of community services GDS-15 the number of illnesses, the ADL and IADL items	Over 30% of older Chinese adults living alone reported at least mild depressive symptoms. Multiple dimensions of social exclusion (like a lower level income adequacy, a higher level of housing condition, and feeling more lonely) were significantly associated with higher depressive symptoms among older Chinese who live alone, with feeling lonely emerging as the strongest predictor.
Hwang and Son Hong (44) Korea	To explore predictors of life satisfaction among older men living alone in South Korea.	**Cross-sectional study** The raw data from the 2020 NSOK.	*N* = 548 *M* = 548 (100%) Aged ≥ 65 years Living alone	**SNI** BMI; NSI checklist; K-IADL 5-item physical performance scale; the 2020 NSOK self-reported questionnaires (containing vision/hearing difficulties, and life satisfaction) GDS-15; MMSE;	Income lower than the standard median income (OR = 2.06, 95% CI = 1.34–3.18), being unemployed (OR = 1.59, 95% CI = 1.06–2.37), living in a rural area (OR = 1.55, 95% CI = 1.01–2.36), nutritional risk (OR = 1.98, 95% CI = 1.34–2.92), declined sensory function (OR = 3.29, 95% CI = 2.20–4.93), depression (OR = 1.86, 95% CI = 1.03–3.35), and **SI** (OR = 1.58, 95% CI = 1.05–2.37) were found to be significant predictors of lower life satisfaction among older men in South Korea.
Melchiorre et al. (36) Italy	To explore the available social support resources for frail older people with functional limitations aging in place alone, and possible links between their SI and perceived loneliness.	**Mixed-method research** Purposive sampling and not probabilistic sampling (quantitative/qualitative). From May to December 2019	*N* = 120 *M* = 30 (25.0%) *F* = 90 (75.0%) Aged ≥ 65 years Living alone: 78%, PCA(personal care assistant): 22% Living alone at home without cohabiting family members, or with a PCA; and absence of help by close family members.	**SI:** asking who do you confide any intimate concerns with the family, friendship and neighborhood networks, and who do you ask for practical or psychological support, or just for talking. Also, asking the methods and frequency of the meetings and contacts with them. **S**ocial support: asking “Please, tell us about who helps you the most among relative, friend, neighbor, service operator”; “Approximately, how often does this person help you in a week?”; “How far away does he/she live?”. **P**erceived loneliness: asking “Do you feel alone/abandoned?”; “How much does it seem that others are attentive to what happens to you?” ADLs-IADLs	The relationship between SI and loneliness emerging from our results do not indicate a clear increasing or decreasing trend, or a linear link, i.e., higher loneliness and lower number of supportive family confidants or vice versa. Thus, loneliness may or may not be associated to a lack of confidants.
Kwan and Tam (37) China, Hong Kong	To explore the dynamics of poverty across the lifespan of older adults-headed households, including older people living alone and those living only with a spouse.	**Qualitative study** Life history interview 2020	*N* = 47 *M* = 10 (21.28%) *F* = 37 (78.72%) Aged ≥ 65 years Living alone or living only with a spouse and in poverty in Hong Kong.	Semi-structured interviews Thematic analysis to identify themes	Thematic analysis was used to identify eight themes related to challenges: (i) SI and loneliness, (ii) self-esteem and self-efficacy, (iii) declining mobility, health and activity levels, (iv) high medical expenses, (v) age discrimination and long wait times for medical health services, (vi) age discrimination, retirement, and wanting part-time employment, (vii) not enough gender-specific social participation activities, and (viii) housing insecurity. Four themes related to strengths were identified: (i) An “I have enough” mindset, (ii) strong formal social support, (iii) contributing to the community and others, and (iv) “Most of us like to be alone.”
Soulières and Charpentier (38) Canada	To discuss the subjective experiences of SI among older people living alone and the ways it can impact older people's relationships.	**Qualitative study** From 2016 to 2019	*N* = 43 *M* = 11 (25.58%) *F* = 32 (74.42%) Age range: 65–93 Living alone in the Montreal area of Canada.	Semi-structured interviews Thematic analysis to identify themes	The majority of the participating older people did not see living alone as a problem. Their stories revealed the extent of their resilience and their ability to maintain satisfactory social relations with family and peers. However, for a minority, mostly men over 80 years old, solo living translated into being alone and could become problematic.

LSNS-6, the Lubben social network scale; SESN, the scale of the older adults self-neglect; family APGAR, the family adaptation, partnership, growth, affection, and resolve index; AAQ, attitudes to aging questionnaire; ULS-6, the 6-item UCLA loneliness scale; GDS-15, the 15-item geriatric depression scale-short form; SPMSQ, short portable mental status questionnaire; Lawton & Brody ADL, the activities of daily living scale of Lawton & Brody (14 items) are grouped into two parts: the physical self-maintenance (PSM) scale (six items, including bathing, feeding, dressing, grooming, toileting, and transferring), and the instrumental activities of daily living (IADL) scale (eight items including washing clothes, using a telephone, cooking, using public transportation, shopping, handling one's own money, doing housework, and taking medicine); ICOPE, the WHO integrated care for older people guideline, including SPPB, self-reported vision and hearing loss, GDS-15, MMSE, MNA-SF; SPPB, the short physical performance battery test; MMSE, mini-mental state examination; MNA-SF, the mini-nutritional assessment short-form CPSS, the Chinese perceived stress scale; HPLP-C, the health-promoting lifestyle profile-Chinese; WHOQOL-OLD, the World Health Organization quality of life—OLD; FRAIL scale, including 5 items such as fatigue, resistance, ambulation, illnesses, loss of weight; GDS-5, geriatric depression scale-5; SRH, self-rated health; IADL, instrumental activities of daily living; ZSDS, the Zung self-rating depression scale; the ADL and IADL items, ADL (four items) including personal self-care such as taking a bath, grooming, eating and drinking, and using the toilet. IADL (eight items) including indicating light house chores, heavy house chores, house maintenance, transportation, shopping, cooking, handling personal business (e.g. paying bills), and using the telephone; SNI, Berkman's social network index, including: religious activity frequency, group/club participation, number of close contacts (3 items); NSI checklist, the nutrition screening initiative checklist; K-IADL, Korean versions of instrumental activities of daily living (10 items); 5-item physical performance scale; the 2020 NSOK self-reported vision difficulties, hearing difficulties, and life satisfaction questionnaires; ADLs-IADLs, 12 Basic and Instrumental Activities of Daily Living: ADLs (six items) are the following: getting into/out of bed, sitting/rising from a chair, dressing/undressing, washing hands and face, bathing or showering and eating/cutting food. IADLs (six items) include the following: preparing food, shopping, cleaning the house, washing the laundry, taking medication in the right doses and at the right times and managing finances.

### 3.2 Quality of studies

The overall quality of the most articles was considered high. The included articles met 80% to 100% of the MMAT criteria. In quantitative studies (comprising 1 cohort study and 10 cross section studies), the response rate ranged from 76.50% (34) to 98.8% (8, 35). However, the quantitative phase of a mixed-method study reported response rates of only 58%, which resulted in its quality being moderate (36). Additionally, the two qualitative studies were evaluated as of high quality (37, 38). All the eligible articles were included regardless of the methodological quality. The MMAT (2018) quality appraisal results for the included qualitative studies are summarized in Table 4.

**Table 4:** MMAT (2018) quality appraisal of the included studies.

Included study	MMAT appraisal criteria																							Overall quality
	Design	S1	S2	1.1	1.2	1.3	1.4	1.5	3.1	3.2	3.3	3.4	3.5	4.1	4.2	4.3	4.4	4.5	5.1	5.2	5.3	5.4	5.5	
Jia et al. (35)	Cross-sectional study	Y	Y											Y	Y	Y	Y	Y						High
Jia et al. (8)	Cross-sectional study	Y	Y											Y	Y	Y	Y	Y						High
Jia et al. (18)	Cross-sectional study	Y	Y											Y	Y	Y	Y	Y						High
Gu et al. (39)	Cross-sectional study	Y	Y											Y	Y	Y	Y	Y						High
Guo et al. (40)	Cross-sectional study	Y	Y											Y	Y	Y	Y	Y						High
Su et al. (41)	Cross-sectional study	Y	Y											Y	Y	Y	Y	Y						High
Xie et al. (42)	Cross-sectional study	Y	Y											Y	Y	Y	Y	Y						High
Imamura et al. (20)	Longitudinal study	Y	Y						Y	Y	Y	Y	Y											High
Imamura et al. (43)	Cross-sectional study	Y	Y											Y	Y	Y	Y	Y						High
Tong et al. (34)	Cross-sectional study	Y	Y											Y	Y	Y	Y	Y						High
Hwang and Son Hong (44)	Cross-sectional study	Y	Y											Y	Y	Y	Y	Y						High
Melchiorre et al. (36)	Mixed-method research	Y	Y	Y	Y	Y	Y	Y						Y	CT	Y	N	Y	Y	Y	Y	CT	N	Moderate
Kwan and Tam (37)	Qualitative study	Y	Y	Y	Y	Y	Y	Y																High
Soulières and Charpentier (38)	Qualitative study	Y	Y	Y	Y	Y	Y	Y																

Response options were standardized as: Y, yes; N, no; CT, can't tell; Screening questions: S1. Are there clear research questions? S2. Do the collected data allow to address the research questions? Qualitative study: 1.1. Is the qualitative approach appropriate to answer the research question? 1.2. Are the qualitative data collection methods adequate to address the research question? 1.3. Are the findings adequately derived from the data? 1.4. Is the interpretation of results sufficiently substantiated by data? 1.5. Is there coherence between qualitative data sources, collection, analysis and interpretation? Quantitative non-randomized study: 3.1. Are the participants representative of the target population? 3.2. Are measurements appropriate regarding both the outcome and intervention (or exposure)? 3.3. Are there complete outcome data? 3.4. Are the confounders accounted for in the design and analysis? 3.5. During the study period, is the intervention administered (or exposure occurred) as intended? Quantitative descriptive study: 4.1. Is the sampling strategy relevant to address the research question? 4.2. Is the sample representative of the target population? 4.3. Are the measurements appropriate? 4.4. Is the risk of non-response bias low? 4.5. Is the statistical analysis appropriate to answer the research question? Mixed methods study: 5.1. Is there an adequate rationale for using a mixed methods design to address the research question? 5.2. Are the different components of the study effectively integrated to answer the research question? 5.3. Are the outputs of the integration of qualitative and quantitative components adequately interpreted? 5.4. Are divergences and inconsistencies between quantitative and qualitative results adequately addressed? 5.5. Do the different components of the study adhere to the quality criteria of each tradition of the methods involved?

### 3.3 Results and synthesis of findings

#### 3.3.1 Standardized validated network scales

Seven cross-sectional studies utilized the Lubben Social Network Scale-6 (LSNS-6) as the primary instrument for measuring social isolation among empty-nest or living-alone older adults in China.

Regarding mental health outcomes, three studies analyzed the mechanisms of action between social isolation and self-neglect from different perspectives. Jia et al. (35) linked the higher levels of social isolation to the high overall self-neglect and the high emotional self-neglect. In addition, negative aging attitudes (8) and loneliness (18) were the mediator between social isolation and self-neglect. Furthermore, in the domain of depression, social isolation also exerts negative impacts on depression, particularly among older adults living alone, who face over 2 times greater risk of developing incident depression (39).

Three studies examined comprehensive health indicators. Two of them revealed the socially isolated empty-nest older adults are more likely to predict lower levels of intrinsic capacity (IC) (40, 41). Besides, perceived stress and health-promoting behaviors were chain meditators between social isolation and IC (41). While another study showed that social isolation was an independent negative predictor of quality of life among community-dwelling oldest-old adults living alone (42).

#### 3.3.2 Objective contact frequency criteria

Two Japanese studies defined SI as contact with non-cohabiting family, relatives, friends, or neighbors less than once per week.

In a prospective longitudinal study, focusing on the physical health domain, it indicated the socially isolated participants who are living alone had the higher all-cause mortality risk than those who were not living alone, and those living alone but without social isolation did not exhibit an increased mortality risk (20).

In a large cross-sectional analysis, focusing on the mental health domain, it found that social isolation was significantly associated with an increased risk of cognitive impairment regardless of living alone status (43).

#### 3.3.3 Custom or adapted measures

Two studies utilized customized or adaptive measurement tools to assess social isolation or related constructs. Both studies examined the associations between social isolation and mental health outcomes. Tong et al. (34) adopted a multidimensional social exclusion framework (dimensions of income adequacy, housing conditions, civic participation, and social relations) among Shanghai older adults living alone. The social relations dimension included social isolation and subjective loneliness. After controlling for demographic and health variables, only lower income adequacy, poorer housing conditions, and higher loneliness were significantly positively associated with depressive symptoms, while social isolation and civic participation showed no significant associations. Hwang et al. (44) adapted the Berkman Social Network Index (SNI), which includes participation in religious/church activities, group activities, and close contacts but excludes marital status, to assess social isolation among Korean older men living alone. The results showed that social isolation was an independent significant predictor of lower life satisfaction.

#### 3.3.4 Qualitative narrative

The final three studies employed one mixed-method study and two qualitative methods to examine the subjective experiences of social isolation among older adults living alone. The findings of the mixed-method study revealed that these frail older adults aging in place alone commonly experienced a contraction of social networks and reduced contact frequency, resulting in insufficient daily living support and diminished subjective well-being (36). Furthermore, loneliness and social isolation were correlated, but there is not a clear increasing or decreasing trend, nor a linear link between them. Similarly, the two final qualitative studies revealed social isolation worsens comprehensive health and well-being among older adults living alone. One highlighted fears of unnoticed death and daily loneliness and isolation, reduced mobility, high medical costs, and age discrimination in healthcare, low self-esteem and fear of isolation among poor solitary older adults (37). The other noted most of Canadian older adults living alone demonstrate resilience, whereas a minority face heightened risks of social isolation due to financial and health constraints that limit their access to resources and autonomy, potentially compromising their overall well-being (38).

## 4 Discussion

The analysis of the 14 studies initially provided evidence supporting the negative impacts generated by social isolation on the physical and mental health of the empty-nest older adults population. However, the definition and operationalization of social isolation were not entirely consistent across the included studies. Variations in conceptualization and measurement may have influenced the interpretation and comparability of associations with physical and mental health outcomes. Nevertheless, the overall pattern of findings was broadly consistent, with most studies indicating adverse associations between social isolation and health outcomes. Indeed, the combination of physiological aging and the changes in living arrangement has impacted the health of older adults around the world (12, 45–47). Social isolation significantly increases all-cause mortality risk in empty-nest older adults individuals and affects their mental health through mechanisms such as self-neglect, loneliness, and depression. Furthermore, the absence of social networks may diminish intrinsic capacity (IC) and quality of life. These findings underscore the vulnerability of the empty-nest older adults population under social isolation, akin to studies on older adults isolation during the pandemic (48), but with greater emphasis on the long-term effects of empty-nest living.

### 4.1 Mental health

Across all SI measurement approaches, SI shows a robust association with adverse mental health outcomes, and the effect strength varies slightly with the operationalization method.

Studies using the LSNS-6 show that higher levels of social isolation are significantly associated with higher overall self-neglect (OR = 3.596, 95% CI: 1.979–6.532) and higher emotional self-neglect (OR = 2.268, 95% CI: 1.27–4.051) (35), while loneliness and aging attitudes partially mediate the relationships between social networks/isolation and self-neglect, with mediation effects accounting for 37.22% (18) and 28.20% (8) of the total effects, respectively. In the domain of depression, social isolation exerts particularly strong negative impacts among older adults living alone (OR = 2.59, 95% CI: 1.13–5.96) (39). Objective contact-frequency criteria further corroborated SI as a chronic stressor accelerating cognitive impairment irrespective of living arrangement (socially isolated but not living alone: OR = 1.74, 95% CI: 1.29–2.33; socially isolated and living alone: OR = 2.10, 95% CI: 1.46–3.01) (43). Studies using customized measurement tools show some heterogeneity. Tong et al. (34) employing a multidimensional social exclusion framework among Shanghai older adults living alone, found that higher loneliness but not objective social isolation were significantly associated with depressive symptoms. Therefore, this finding suggests that objective deficits in social networks and subjective emotional experiences have important but somewhat distinct effects on older adults' mental health. Hwang et al. (6) using an adapted Berkman Social Network Index among Korean older men living alone, showed that social isolation was an independent significant predictor of lower life satisfaction (OR = 1.58, 95% CI: 1.05–2.37). These studies highlight social isolation as a global threat to the mental health of empty-nest older adults. From intrinsic mechanisms, social isolation may stimulate sustained elevation of stress hormones (e.g., cortisol) (49, 50), and physiological functional decline (51, 52), leading to significantly enhanced loneliness and depressive moods, and potentially accelerating neurodegenerative changes, resulting in evident cognitive impairment. From extrinsic mechanisms, social isolation can reduce social support (53, 54) and participation (55), further amplifying tendencies toward loneliness, depression, and self-neglect, worsening aging attitudes (56), and thereby significantly lowering life satisfaction and overall quality of life. In summary, social isolation accelerates decline at the physiological level and disrupts protective factors at the psycho-social level, forming a vicious cycle. Collectively, these results indicate that intervention strategies should address both objective social networks and subjective emotional experiences.

### 4.2 Physical health

Previous studies indicate that social isolation is a risk factor affecting the physical health of the older adults population (57), which has been emphasized in multi-regional studies. Meanwhile, a cohort study shows a significant association between social isolation and all-cause mortality in older adults (HR: 1.17, 95% CI: 1.09–1.27, *p* < 0.001) (58). However, in this integrated review, evidence on physical health outcomes remains relatively limited, primarily from one Japanese prospective longitudinal study. Social isolation is associated with increased all-cause mortality risk in older adults, regardless of living alone status. However, the combination of social isolation and living alone exhibits the highest mortality risk (HR = 2.08, 95% CI: 1.08–4.00), exceeding that for social isolation without living alone (HR = 1.41, 95% CI: 0.90–2.20) or living alone without social isolation (HR = 0.81, 95% CI: 0.44–1.49) (20). Plausible mechanisms include social isolation elevating stress hormones (such as cortisol) through activation of the hypothalamic-pituitary-adrenal (HPA) axis (49), thereby inducing inflammatory responses and immune function decline (51, 59), leading to increased cardiovascular events (such as myocardial infarction) and heightened mortality risk. Additionally, social isolation and loneliness can impair medication adherence in older adults, thereby exacerbating mortality threats (60).

### 4.3 Comprehensive health and well-being

Our findings also indicate that social isolation exhibits a negative correlation with comprehensive health indicators, such as intrinsic capacity and quality of life. For instance, research on intrinsic capacity among community-dwelling empty-nest older adults in China reveals that individuals experiencing high levels of social isolation demonstrate lower intrinsic capacity (40, 41), driven by chain mediation through heightened perceived stress and diminished health-promoting behaviors (41). In the context of living-alone empty-nest older adults, social isolation emerges as a significant predictor of diminished quality of life (42). This mixed-methods study and 2 qualitative studies further enriched these findings. The result of mixed-methods study showed that frail older adults aging in place alone commonly experienced social network contraction and reduced contact frequency, resulting in insufficient daily living support and diminished subjective well-being (36). Meanwhile, loneliness and social isolation were correlated, no clear linear or directional trend was evident (36). The two qualitative studies similarly demonstrated that social isolation worsens comprehensive health and well-being. One of them, focusing on living-alone empty-nest older adults, uncover associations between social isolation and a range of adverse outcomes, including feelings of loneliness and fear, apprehensions about dying unnoticed, mobility restrictions, healthcare discrimination, and low self-esteem (37). The other noted that while most Canadian older adults living alone exhibit resilience, a minority who are socially isolated face heightened risks due to financial and health constraints that limit resource access and autonomy, potentially compromising overall well-being (38). The negative impacts of social isolation on older adults' comprehensive health can be explained through multiple intertwined mechanisms. First, it directly accelerates physical function decline while disrupting healthy lifestyles (22), thereby diminishing intrinsic capacity (61) and further exacerbating somatic health impairments. Second, social isolation is strongly associated with negative emotions (62). These emotions impair quality of life and subjective well-being directly (63) and, through psychophysiological interactions (49), weaken cognitive function, immune responses, and cardiovascular health, ultimately forming a vicious cycle (52, 59). Collectively, these proofs consistently demonstrate that social isolation imposes multifaceted negative impacts on comprehensive health of empty-nest older adults, encompassing bodily functions, psychological states, and holistic well-being.

### 4.4 Limitations

This integrative review has some limitations that should be highlighted. First, the populations in most of the included studies are from Asia, particularly China, which limits generalizability to other regions with different cultural, socioeconomic, or healthcare contexts. Second, the majority of studies employed cross-sectional designs, which restrict causal inferences, while qualitative studies were underrepresented, potentially overlooking nuanced personal experiences. Third, the use of multiple and heterogeneous measurement tools for social isolation (e.g., LSNS-6, objective contact frequency criteria, and customized instruments) across the included studies introduced substantial heterogeneity, which may affect the comparability and synthesis of findings. Fourth, the focus was largely on solitary empty-nest older adults with limited attention to those cohabiting with spouses, thus underrepresenting diverse living arrangements and their impacts on social isolation.

### 4.5 Future research

Future research can be conducted from the following aspects:

(1) Increase studies on empty-nest older adults in regions such as Europe and America, Latin America, and Africa, and perform cross-cultural comparisons to expand geographical diversity, identify cultural differences, and enhance the universality of findings. (2) Adopt more qualitative research, longitudinal designs, and mixed methods to address the causal limitations of cross-sectional studies and provide deeper individual perspectives. (3) Subdivide the empty-nest older adults population, including those without children, living alone, and living with spouses, compare the impacts of social isolation under different living arrangements, and explore the roles of spouse support and other factors. (4) In addition, integrate digital tools (such as virtual network or wearable devices) to conduct intervention studies and develop strategies to alleviate the negative effects of social isolation on physical and mental health.

## 5 Conclusion

Social isolation among empty-nest older adults, often living alone, is associated with adverse physical and mental health outcomes, including increased mortality, self-neglect, diminished intrinsic capacity, cognitive impairment, and reduced quality of life, accompanied by heightened depression, loneliness, anxiety, and low life satisfaction. Mediating factors such as aging attitudes, loneliness, perceived stress, and health-promoting behaviors exacerbate these effects. Based on the findings, it is imperative to urge policymakers, healthcare professionals, researchers, and educators to develop interventions that promote social support networks and expand social connections to enhance the physical and mental health of empty-nest older adults.
